# Full Spectrum Raman Excitation Mapping Spectroscopy

**DOI:** 10.1038/s41598-020-65757-9

**Published:** 2020-06-08

**Authors:** Paul Finnie, Jianying Ouyang, Jacques Lefebvre

**Affiliations:** National Research Council Canada, 1200 Montreal Road, Ottawa, Ontario K1A 0R6 Canada

**Keywords:** Raman spectroscopy, Carbon nanotubes and fullerenes, Raman spectroscopy

## Abstract

A generalization of the Raman scattering (RS) spectrum, the Raman excitation map (REM) is a hyperspectral two-dimensional (2D) data set encoding vibrational spectra, electronic spectra *and their coupling*. Despite the great potential of REM for optical sensing and characterization with remarkable sensitivity and selectivity, the difficulty of obtaining maps and the length of time required to acquire them has been practically limiting. Here we show, with a simple setup using current optical equipment, that maps can be obtained much more rapidly than before (~ms to ~100 s now *vs*. ~1000 s to hours before) over a broad excitation range (here ~100 nm is demonstrated, with larger ranges straightforward to obtain), thus taking better advantage of scattering resonance. We obtain maps from different forms of carbon: graphite, graphene, purified single walled carbon nanotubes (SWCNTs) and chirality enriched SWCNTs. The relative speed and simplicity of the technique make REM a practical and sensitive tool for chemical analysis and materials characterization.

## Introduction

**Figure 1:**
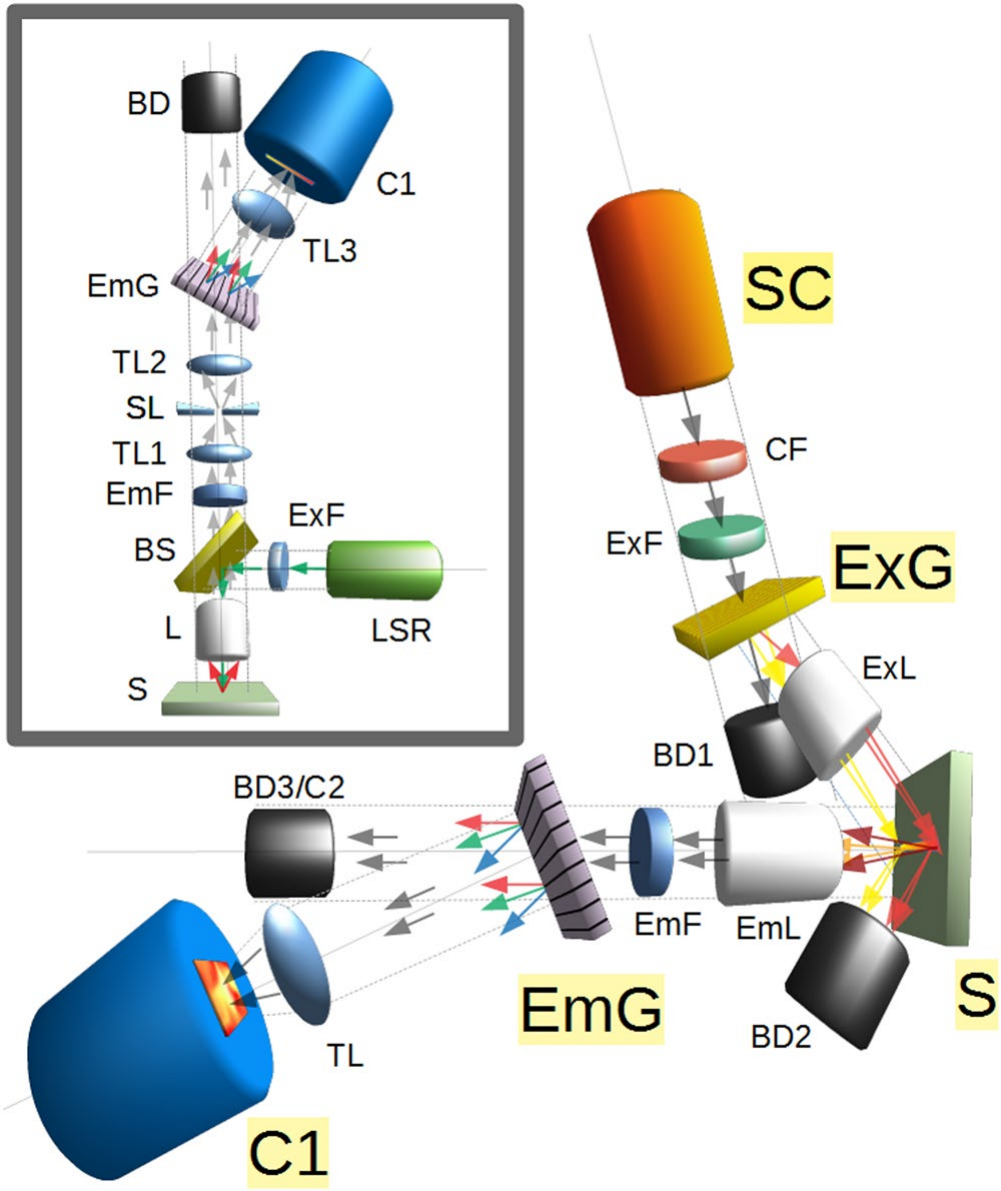
Full Spectrum Raman Spectroscopy Setup. Arrows show the intended path of light. White supercontinuum (SC) light is filtered (CF, ExF) and angularly color separated by a transmission grating (ExG, top beamline).The light is then focused by a microscope objective lens (ExL) into a color graded vertical line on the sample (S). This light is collected by a microscope objective lens (EmL), filtered (EmF) and diffracted angularly by a transmission grating (EmG) in horizontal plane. This is imaged by tube lens (TL) onto a multi-megapixel CMOS camera (C1). Beam dumps (BD1, BD2, BD3) collect unwanted light. A second camera (C2) is used to image the zero order (undiffracted) image. The inset shows a standard micro-Raman setup for comparison. A monochromatic laser (LSR) is used as light source, a beam splitter (BS) is ordinarily used (optional) so that the two objectives (EmL, ExG) can be combined into one (L). It is also common to use a slit (SL). The grating (EmG) is shown in a transmission spectrograph configuration with two tube lenses (TL2, TL3). Additional details are in the Methods.

RS spectra are one dimensional (1D) plots of intensity versus wavelength shift providing a fingerprint for chemical analysis^1^ (including chemometrics^2^), widely applied to nanocarbons^3^, and many other sample types. In RS, incident monochromatic light is scattered by phonons to produce peaks shifted by the phonon frequency. In micro-RS, a microscope objective focuses light onto a sample, elastically (Rayleigh) scattered light is filtered out, and the scatter is analyzed (inset, Fig. 1). This is usually limited to a single laser wavelength (λ), or a few discrete wavelengths.

However, the precise choice of λ is consequential for the signal intensity because it has non-resonant RS and resonant RS (RRS) components^4^. Scattering efficiencies can rise by orders of magnitude (~10^3^–10^8^×) for λ near a resonance. The intensity of a Raman mode *vs*. λ is its resonant excitation profile (REP)^5^. Peaks in the RS spectra are vibrational, while features in the REP are electronic in origin. Plotting the intensity versus Raman shift and λ makes a REM, with horizontal slices that are RS spectra and vertical slices that are REPs. Ordinarily, to obtain a REM, many laser wavelengths are taken sequentially with a tunable laser to build up a two-dimensional (2D) map^6,7^.

While these techniques are very general, RRS (and REM) is particularly important for nanocarbons^3,8^. For SWCNTs, the precise chemical structure is linked to the optical spectrum by the Kataura^9^ plot, with which RRS can be used to identify nanotube species^10^. Such data helps detect trace metals and qualify/quantify the metal/semiconducting purity in enriched SWNCT materials, an issue in nanoelectronics^6,11,12^. In spite of its proven analytical value, because it is slow and technically cumbersome^6,13,14^ REM is not widely used, even for nanocarbons.

The proposed hyperspectral “full spectrum” approach makes REM rapid and relatively simple, having no moving parts, and relatively low cost to implement. By “full spectrum” here, we mean this can be done in principle for any color, and that many wavelengths are used all at once. Updating a classic approach to parallelization^15^, this is akin to “line illumination” RS^1^ but rather than illuminating monochromatically, a color gradient is used. Recent photoluminescence (PL) excitation experiments use such an approach^16,17^. Challenges for using such methods for RS as opposed to PL include the weak signal (~10^−6^ *vs*. ~10^−1^ efficiency for PL^18^), the strong, unwanted Rayleigh background, the need for higher (~10×) spectral resolution, the potential for complications due to laser heating, and the more complicated data processing that is required. All these challenges are overcome here with current generation optical components.

It is technically difficult and costly to span a large range of excitation wavelengths for RS with conventional monochromatic lasers which are ordinarily wavelength tuned for REM. Instead, here a supercontinuum (SC) light source provides broadband (~450 nm–2200 nm) light for all wavelengths, all at once. Unlike other broadband sources, SC has laser-like collimation and so can be tightly focused and otherwise manipulated, but it still has good spectral power density (~0.1 mW/cm^−1^). Some important steps have been demonstrated with broadband light sources. Recently, SC has been used to map graphene by stepping serially in time^19^. Also, notably, (non-SC) diode illumination has been spectrally dispersed in a line to obtain 1D RS spectra^20^. Here, SC is nearly monochromatic in a spatially compact spot (~10 μm), and spatially dispersed (~mm scale) to cover a large excitation bandwidth (here ~100 nm), so that spectra are high resolution, but such that sample heating is insignificant. Unlike conventional RRS, an entire range of excitation wavelengths is used simultaneously.

## Methods

### Setup

See Fig. 1 for a schematic illustration of the setup. The supercontinuum light source (SC) was an NKT Photonics SuperK Extreme High Power Super Continuum White Light Laser (EXR-15). The cold filter (CF) stage, depicted here as a single filter, was actually a folded cavity with a visible band high reflecting mirror slightly tilted from normal. This allowed multiple reflections before transmission and so acted as a high rejection bandpass filter, passing the visible light but not the strong NIR which is a source of heat, and background for RS. A short wave pass edge filter designed for anti-Stokes RS spectroscopy at 633 nm (Iridian Spectral Technologies) was used as an excitation filter (ExF). The grating to disperse the exciting white light (ExG) was a 1200 lines/mm holographic transmission grating (Wasatch Photonics). The dispersed excitation light was focused to a line with a 10 × 0.26 numerical aperture long working distance NIR microscope objective (ExL, Mitutoyo). Samples were mounted by taping them to a glass slide and clamping them on a micrometer driven *xyz* stage. Collection was by a 10 × 0.45 numerical aperture long working distance microscope objective (EmL, Edmund Optics). A long wave pass edge filter designed for Stokes Raman spectroscopy at 633 nm was used as an emission filter (EmF, Semrock). A 1200 lines/mm holographic transmission grating (Wasatch Photonics) was used to disperse the scattered light. A 75 mm focal length achromatic lens (Thorlabs) was used as a tube lens (TL). The camera (C1) was a 5.5 megapixel cooled CMOS detector (Andor Neo 5.5CL). The other camera (C2) was a low cost room temperature megapixel webcam (Edmund Optics).

### Materials

The HOPG (Structure Probe, Inc.), graphene (Structure Probe, Inc.), and unsorted SWCNTs (Raymor Nanointegris) were obtained commercially. The HOPG was taped to a glass slide with double sided tape, and a second glass slide with double sided tape was pressed against it to remove the top surface and reveal a clean layer. The graphene sample was supported by silicon substrate which was taped to a glass slide. The unsorted SWCNTs were aqueous suspensions and were drop cast directly onto a glass slide producing a black, optically thick film. The chiral and diameter sorted SWCNTs (6,5), (9,8) and (7,5) were sorted from as-prepared bulk SWCNTs using polymer wrapping procedures (See Supplementary Information for details). Sorted SWCNT dispersions were drop cast on PTFE membranes (0.2 µm pore size) until optically thick films were formed, and were rinsed in toluene to remove the excess polymer. The PTFE membranes were taped onto glass slides for measurement.

### Spectral calibration

The emission wavelength was calibrated with three narrow band edge filters placed at the “EmF” position at 659.2 nm (Alluxa), 706.5 nm (Alluxa) and 752 nm (Iridian Spectral Technologies). The excitation wavelength was calibrated to the band edge of short wave pass filter near 633 nm and by setting the G band RS peak resonance to 1590 cm^−1^.

### Spatial resolution

This instrument did not reach diffraction-limited spatial resolution. For our setup, the illumination line on the sample was wide (~43 μm) and this limits the spatial resolution horizontally – i.e. perpendicular to the illumination line axis. (See Supplementary Information Fig. S1). Parallel to the illumination line, intensity fluctuations as small as ~1 pixel in height were observable (See Supplementary Information Fig. S2). This is 6.5 μm on the spectroscopy camera, which, at the magnification of ~3.8×, corresponds to a spatial resolution of 1.7 μm on the sample surface. This is the limiting factor for the spatial resolution along the vertical axis. With appropriate components, we expect that much higher spatial resolution could be obtained from a similar instrument. For example, using higher numerical apertures and pixel sizes adapted to the focal spot will increase the spatial resolution.

### Spectral resolution

There are two spectral resolutions to consider in REM, the Raman spectral resolution (*x-*axis) and the excitation spectral resolution (*y-*axis). The spectral resolution can be estimated with a spectral line which is narrower than the resolution. The full width at half maximum (FWHM) of G band of HOPG was ~28 pixels wide on the spectroscopy camera, corresponding to ~180 μm on the camera and ~47 μm on the sample. This is similar to the spatial size of the illumination line, providing evidence that the spatial linewidth is setting the spectral resolution of this instrument. (See Supplementary Information Fig. S3 for more detail.) Given the dispersion of ~0.67 pixels/cm^−1^ this corresponds to a spectral resolution of ~41 cm^−1^. This is also the effective limit on the spectral resolution with respect to excitation wavelength.

The specifications of the supercontinuum light beam can limit the spectral resolution. First, the beam diameter is specified at ~1 mm at 530 nm increasing to ~2 mm at 1100 nm. This beam diameter is smaller than microscope objective’s pupil size. So, it is this beam diameter that determines the effective numerical aperture for focusing. The spatial resolution scale *d* is given by the Abbe relation *d* = λ/(2*n* sinθ), with λ the wavelength, *n* the index of refraction and θ the collection angle. Using 0.5 mm beam radius at a working distance of 30 mm from sample to microscope objective gives θ ≈ 1° which, using *n* = 1 for air, gives a resolution of ~17 μm at 530 nm. This is comparable in scale to the experimental resolution, and so may already be a limiting factor here. Steps that would improve this include using a microscope objective with higher numerical aperture/shorter working distance, using a beam expander on the supercontinuum beam, or using a different supercontinuum source that better fills the objective.

A separate consideration is the pulsed nature of the light source. Here, the supercontinuum light source is seeded by a laser with 5 ps duration pulses. Although the supercontinuum light is not by any means a monochromatic pulse, it is instructive to consider how monochromatic the spectrally dispersed light could actually be if it had this same 5 ps pulse duration. A Fourier transform limited pulse has Δτ·Δν = P where P is a pulse shape dependent and is ≈0.4 for Gaussian pulse shapes. For a Δτ = 5 ps Gaussian pulse then, Δν = 80 GHz. At 633 nm (474 THz), 80 GHz corresponds to 0.11 nm. In cm^−1^, 633 nm corresponds to 15798 cm^−1^, so that results in a spectral resolution of ~3 cm^−1^. This would be a best case resolution spectral resolution for a 5 ps Gaussian monochromatic pulse. Although the spectrally dispersed visible light from the supercontinuum is far removed from this simple situation, it does suggest that the spectral resolution could be limited to ~ a few cm^−1^ due to the pulsed nature of the beam. If this is the case, the spectral resolution can be improved by using a source with a longer pulse duration, or, ideally, a continuous wave (cw) source.

### Spectral bandwidth

Since the supercontinuum light is extremely broadband, what determines the wavelength range is the dispersion of the excitation grating (ExG) and the size of the excitation microscope objective entrance pupil, or any other such obstruction in the optical path. Physically, the grating disperses the collimated white light supercontinuum beam, converting wavelength into angle. The range of wavelengths in the illumination line is set geometrically by this angle, the diameter of the beam, and the size of the entrance pupil going into the microscope objective. The smaller the grating dispersion the larger the bandwidth, and the larger the entrance pupil, and the closer it is to the grating, the larger the bandwidth. Higher magnification objectives tend to have smaller entrance pupils, so spatial resolution is reduced if bandwidths are increased by increasing entrance pupil diameters. The instrument we demonstrate here has a fairly high grating dispersion, and a fairly large entrance pupil. Both the grating dispersion and the entrance pupil can be made larger or smaller, if desired, with off the shelf optical components. Lower dispersion gratings lead to smaller angles, and so higher bandwidth, but lower dispersion gratings have lower spectral resolution, so there is the usual trade-off between resolution and bandwidth in grating spectrometers if the grating dispersion is reduced to obtain a larger range of wavelengths.

### Wavelength dependence of intensity

Any optical system will have some wavelength dependence. For the most part, the wavelength dependence of this system is gradually changing with wavelength, so any significant changes in intensity here can be attributed to the sample, except, obviously, near the cut-on or cut-off edges of the interference filters. The supercontinuum source, camera, and gratings have the most variation with wavelength, but all are quite gradual. The supercontinuum light intensity primarily increases gradually with increasing wavelength, and is evaluated explicitly below. The camera specifications give a quantum efficiency near 60% at 600 nm dropping to ~33% at 800 nm. The detection grating specifications have the opposite trend, with a smoothly varying efficiency curve near ~60% efficiency at 700 nm and ~85% efficiency at 800 nm. The interference filters have transmissions specified as better than 93% and so only introduce minor ripple effects (a few %) away from their cut-off wavelengths. The result of all this is that REMs we obtain are meaningful, even without any calibration, keeping in mind these gradual, smaller variations, and the filter cut-offs.

The rigorous calibration of Raman scattering intensity as a function of Raman shift is not simple, even for standard, monochromatic laser Raman spectroscopy. Procedures for calibrating conventional monochromatic laser Raman spectra are described in detail in ref. ^1^ and codified into standard protocols as described in ref. 21 The reason for the complexity is not only that the collection optical system will have some wavelength and polarization dependence, but also because Raman scattering matrix elements depend on the spatial orientation with respect to the polarization of light. In fact, all aspects of the metrology of the calibration of Raman spectra continue to be actively developed by standards organizations such as the Versailles Advanced Materials and Standards Organization (VAMAS) in its technical working area (TWA) 42 “Raman Spectroscopy”^22^.

As indicated in ref. ^1^, and still the case now, most published Raman spectra are not corrected for the instrumental response. To make such calibrations, the protocols described in ref. ^21^ use classic optical spectroscopic methods of “standard candles” where either a known, calibrated light source (e.g. blackbody of fixed temperature) or a substrate with a known fluorescence spectrum (e.g doped glass) is measured by the optical system. The wavelength dependent response of the detection system is determined from the ratio of the known signal and the measured signal. This ratio is applied as a factor (by assuming linearity) to subsequently measured data to obtain Raman spectra with calibrated relative intensity as a function of wavelength.

These approaches can be extended to REM with the complication that the laser intensity (either in power or photon incident rate) is not generally constant as the wavelength is changed. For conventional REM, with laser wavelength tuned serially in time, the laser power can be actively controlled to maintain fixed power (or fixed photon/rate), or the laser power can be allowed to vary with wavelength and the spectra can be normalized with the assumption of linear scaling of scattering intensity with the incident photon rate - ordinarily a good approximation when the laser power is low. Similar monitoring of the laser power at all wavelengths could be accomplished here by directing part of the beam or spectrally dispersed Rayleigh scattering to a spectrometer or spectrum analyzer. There is a more current discussion of intensity calibration by such methods in tunable laser Raman excitation mapping (for lasers tuned serially in time) in refs. ^5,6^.

Here, with a “full spectrum” approach, intensity calibration is a particular challenge because two dimensions - excitation and collection - each with their own variations must be accounted for together, at the same time. The detection side will have an instrumental spectral response, which ideally would not - but possibly could - vary spatially across the focal plane of the camera. In addition, the supercontinuum light source varies in intensity with wavelength. Therefore it is simpler here, and probably more common in REM in general, to use a known Raman spectrum as a benchmark and compare intensities of the test sample to the known Raman spectrum. This is different from the standard procedures of ref. ^21^ where the samples have certified fluorescence spectra when illuminated by a single, specific laser wavelength. The fluorescence peak covers only a fairly limited range of wavenumbers and its shape is not generally the same, or certified for other wavelengths.

As opposed to using fluorescence or illumination “standard candles”, a good approach is to measure a known *non-resonant* Raman scatterer and obtain its REM, and use this known spectrum to calibrate the variation with laser power. Non-resonant Raman scattering intensities have the characteristic ~1/λ^4^ scattering dependence of wavelength (in units of power). So, if one has a Raman scatterer that is known to be non-resonant, the evolution of the spectral lines over the map can be corrected by a multiplication factor at each point to reproduce the expected ~1/λ^4^ non-resonant REP. A strength of this approach is its practical simplicity, as it relies only on measuring one extra sample or compound to obtain the correction factors. A weakness is that the response is only rigorously measured at the Raman bands, not over the entire map, so it works best for reference compounds with many spectral lines, and depends on having a sufficiently smoothly varying collection optics side spectral response.

Here, we could correct these SWCNT spectra by dividing through by such correction factors derived from the HOPG or graphene map intensities. However, those maps are noisy, and so would more ideally be integrated longer. Also HOPG and graphene have relatively few spectral lines, although fortunately they are very close to some of the SWCNT lines of interest. So, we prefer not to explicitly make a correction like this, but rather we prefer to simply use the HOPG and graphene peak intensities as a guide to give us a good idea how much impact variations in illumination power and instrument response have on the spectra. Since the illumination and detection efficiencies are the same independent of sample - they all are subject to the same instrumental efficiencies - it is meaningful to compare different maps for different samples. Resonant structure can be confirmed in the SWCNT samples by comparing to the more gradually evolving HOPG or graphene intensities. The HOPG G band is an excellent benchmark for comparing to the SWCNT G bands.

As an extra check on the meaning of the intensity variation in these maps, the wavelength dependence of the illumination intensity was also measured separately. (See Supplementary Information, Fig. S4) The intensity, in counts, increased with wavelength, roughly by a factor of two over the scan range, similar to a blackbody source but with an increase just before the cut-off wavelength (633 nm). Therefore, if illumination intensity were the only factor, the scattering intensity would increase with wavelength. Of course, the non-resonant Raman scattering would have the ~1/λ^4^ wavelength dependence, which would lead to a ~30% reduction in scattering probability over a change of ~50 nm in the visible range.

In the future it will be important to develop rigorous and practical intensity calibration procedures for this method, just as is it important in more established methods of Raman spectroscopy.

### Signal-to-noise

To understand the performance of this particular instrument, and the visibility of the RS signal, it is useful to determine the signal-to-noise level. A detailed calculation, made by comparing two datasets from the same area is presented in the Supplementary Information (Fig. S5 and Table S1). For 500 ms acquisitions the most visible and prominent features had a signal to noise ratio (SNR) ratio of 18 and the three strongest SWCNT Raman bands had SNR ratio of 10 or greater. This is the SNR of the equivalent of a monochromatic laser’s horizontal slice in the REM. Here, the incident power was ~10 mW dispersed over the band ~540 nm to ~630 nm. More qualitatively, the brightest bands were still visible and potentially usable for 5 msec integrations, and weak bands were easily visible with longer integrations, as might be expected. (Supplementary Information Fig. S6).

### Data processing

Acquisition was with Andor Solis software, using rolling shutter mode, and no binning. One minute integrations were taken by summing six 10 second integrations. (Integration times as short as 5 ms are demonstrated in the Supplementary Information Fig. S6) Images were converted to Ascii format. The data was imported using Pandas into Python 3. Data was manipulated with the numpy package and plotted using matplotlib. A constant base-plane background level has been subtracted from all maps (i.e. constant regardless of horizontal or vertical position on the map). To convert from horizontal pixel to Raman shift, the intensity at each Raman shift at 2 cm^−1^ intervals from 100 cm^−1^ to 3600 cm^−1^ was calculated by linearly interpolating the intensity measured in counts on the nearest neighbor pixels with calibrated shifts just to the left and right of these values.

Essentially, the signal is recorded by pixel - which maps very nearly to wavelength because of the near-linearity of the grating dispersion - and is then processed into Raman shift. This is stated explicitly only to be clear: The intensity, although calculated at each Raman shift, is an intensity per pixel, which most closely approximates a bin of fixed interval in wavelength, as opposed to a bin of fixed interval in Raman shift. These are, of course, different because Raman shift is an energy, not a wavelength. In other words, there is no adjustment in the intensity for the variation in the bin size of each pixel in terms of Raman shift.

## Results and Discussion

Figure 1 shows a schematic of the setup. Details are in the Methods section. In essence, a SC source is filtered to block unwanted heating and background. A grating disperses the SC light spectrally and a microscope objective focuses it to a color-graded line. A separate objective collects the scattered light, with the excitation angled outside its collection cone. An edge filter blocks the Rayleigh scatter, and a grating angled orthogonally to the first disperses the signal, which is imaged by a camera. So, the vertical axis encodes the excitation wavelength, and the horizontal axis encodes the scattered wavelength.

**Figure 2:**
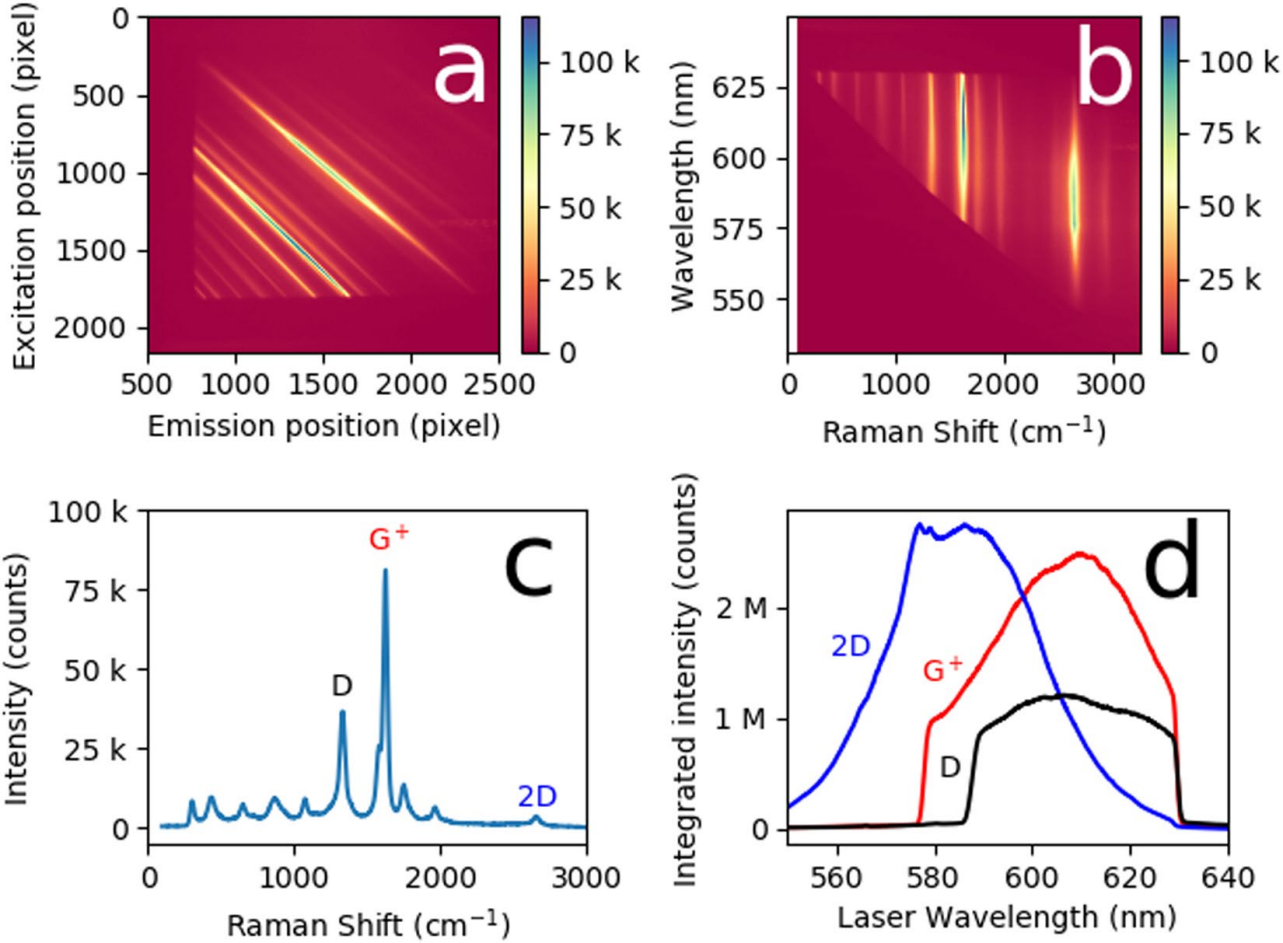
Full Spectrum Raman Excitation Mapping (**a**) unprocessed map as it appears on the CMOS camera pixel array (**b**) the same map transformed into Raman shift and wavelength coordinates (**c**) a Raman spectrum from a single pixel high horizontal slice of the map at 623.3 nm. Three peaks are labelled: D, G^+^, and 2D, but at least nine strong peaks are visible, and can be attributed, in order, to RBM, IFM^−^, oTO, IFM^+^, D, G^−^, G^+^, M, iTOLA and 2D (also called G’) bands (see e.g. ref. ^3^). (**d**) Raman excitation profiles of the D, G^+^ and 2D bands from strips integrating over the entire band. The sharp drop-offs at the left and right extremes are due to the optical filters.

Figure 2 shows the result for a film of (7,5) SWCNTs. (See Methods). The incident power was ~10 mW from ~540 nm to ~630 nm. Peak intensities were ~10^5^ counts/pixel in 60 s. Figure 2(a) shows the real-time camera image. Figure 2(b) shows this transformed to nm (excitation) and shift (cm^−1^). The filters create the triangular shape with a short wave pass excitation filter (horizontal) and a long wave pass (diagonal). Nine strong peaks are visible, and can be identified with tables.^3^ Higher order combination modes area also visible in (a). Figure 2(c) shows a horizontal slice, corresponding to a conventional RS spectrum (623 nm). Figure 2(d) shows vertical slices corresponding to the D, G^+^ and 2D band REPs. This is the integrated intensity of each band, by summing over a bin in Raman shift containing the band.

Importantly, Fig. 2 recovers all the information that one gets from conventional monochromatic laser Raman spectrum. However, since a supercontinuum laser has been used, there is no restriction on the incident light due to the choice of the source (this source emits ~450 nm to ~2 μm), as there is for monochromatic lasers or even for tunable lasers, which generally cover a more limited range. In addition to the Raman spectrum the REPs of many bands can be evaluated simultaneously. Thus, a “full spectrum” REM can be obtained.

This, our first demonstration of the method, uses a simple Rayleigh scattering rejection scheme. That is, we use a single short wave pass filter on the excitation optics and a single matched long wave pass filter on the collection optics side. The result is that the map is restricted to the triangular shape bounded by the cut-off wavelength of the filters. However, with no change to the configuration, the boundaries can easily be changed by swapping edge filters. The edge filters can also be angle tuned, a scheme used in some tunable filter Raman excitation mapping systems^6^. With a single short wave pass/long wave pass filter combination the bandwidth of REPs is small for small Raman shift, and increases as the Raman shift becomes large. So, for example, the 2D band has quite a wide REP while other bands may not.

It must be noted that the ideal sample is flat and homogeneous. If the sample is sufficiently flat and homogeneous, a single acquisition is sufficient to obtain a representative REM. This is easily confirmed experimentally by translating the sample spatially in the plane, while obtaining real-time REMs. So, to obtain the resonant excitation profile (REP) in a single shot we are using spatial homogeneity of the sample. It is true that inhomogeneity of the sample will be reflected in the measured data. However, if desired, it is possible to scan the sample spatially to build up a multidimensional data set that includes REMs of each point on the substrate. However, these samples are sufficiently homogeneous that we have not done so here.

**Figure 3:**
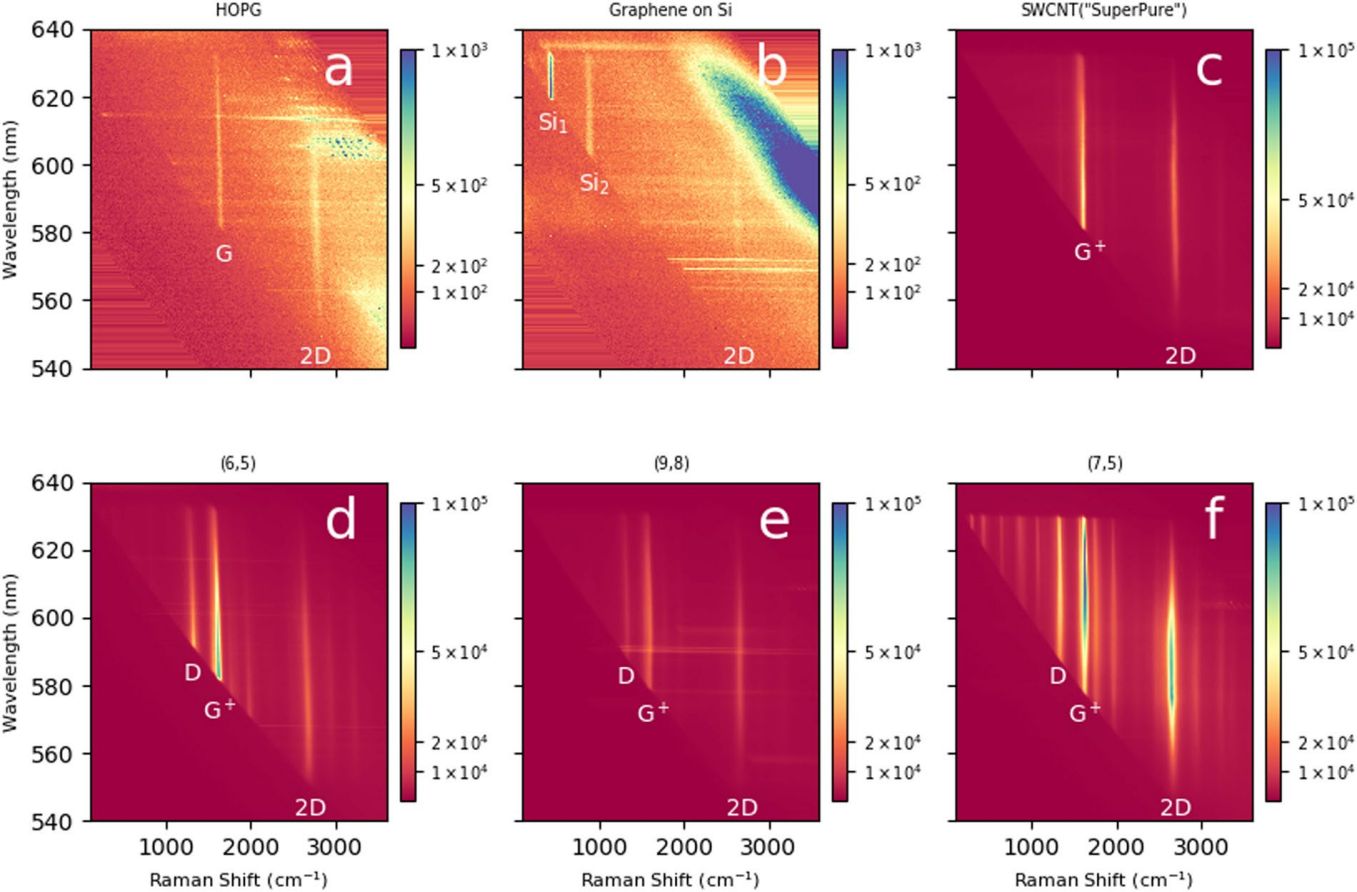
Raman Excitation Maps of Some Carbon Materials (**a**) highly oriented pyrolytic graphite (HOPG), (**b**) graphene on silicon (**c**) commercial unsorted SWCNTs (NanoIntegris SuperPure SWCNT) (**d**) chirality enriched (6,5) SWCNTs, (**e**) chirality enriched (9,8) SWCNTs, (**f**) chirality enriched (7,5) SWCNTs. The SWCNTs are all optically thick films. The color scale for HOPG and graphene extends to 10^3^, while the scale for the SWCNTs is extends to 10^5^, a factor of one hundred higher. For (**a**) graphite, graphitic G and 2D (or G’) bands are labelled. For (**b**) first and second order silicon peaks are labelled (Si_1_, Si_2_), as well as the graphene 2D band. For the SWCNT samples D, G^+^, and 2D (or G’) bands have been labelled, except (**c**) where the D band is not labelled because it is barely visible on this scale.

Figure 3 shows different carbons under identical conditions. Horizontal lines are artifacts of scattering from particulates. The graphite (a) and graphene (b) signals are weak, so the color scale has been magnified 100× relative to the other samples. All samples show a G band (~1590 cm^−1^) – called G^+^ for SWCNTs – and 2D band (~2600 cm^−1^, also called G’). The G band for graphene is imperceptibly weak on this scale. (But it is shown in Supplementary Information Fig. S7). The graphene 2D band is only ~200 counts/pixel, with the strongest peaks coming from the silicon substrate underneath. The intensities for HOPG are ~400 counts/pixel (G and 2D bands). The SC power, camera response, and transmission are wavelength dependent, however they have the same dependencies for all the maps. Also, since the bands for HOPG are flat, they can be used as a benchmark for comparison. (See Methods for a detailed discussion).

The SWCNT films produce much stronger signals. Figure 3(c) shows unsorted SWCNTs, and (d)-(f) show different species, all on the same scale. The unsorted sample has strong G^+^ and 2D bands, but other bands are barely visible. Of the sorted, chirality enriched samples, the (7,5) in (f) is most intense at ~10^5^ counts/pixel (G band), (~250×), greater than HOPG, and broader. The (6,5) sample reaches similar intensities, but the REP is shifted to smaller wavelength, as expected [~566 nm for (6,5) ~645 nm for (7,5)^24^] The (9,8) species, which is far from resonance, is weaker, but it is still much more intense than HOPG (~10^2^×).

**Figure 4:**
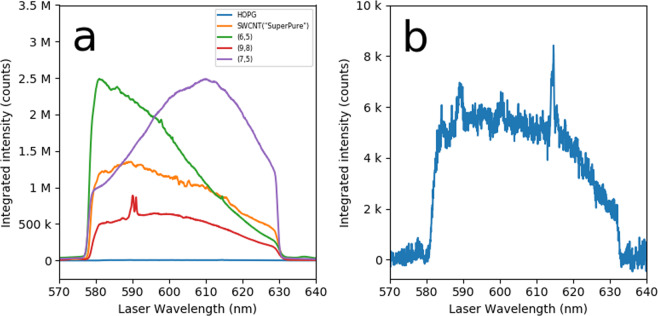
Raman Excitation Profiles of the Integrated G Band Intensity for Different Samples (**a**) The highly oriented pyrolytic graphite (HOPG) sample (blue) is so low relative to the others to be negligible on this scale. Spikes such as those on the red curve at 590 nm are due to scattering from particulates and surface features. (**b**) The HOPG sample on a 350× zoomed *y*-axis scale. The spikes such as those at 588 nm and 615 nm are due to spatial variations in scattering from particulates.

For REPs it is most meaningful to compare similar bands of different samples obtained under identical conditions. This accounts for the wavelength dependence of the instrument, since it is the same for all samples. Figure 4 shows extracted REPs for the G band region of all samples, except graphene which is barely above the noise (See Supplementary Information Fig. S7). These are integrated REPs, obtained from integrating the maps over the entire G band region. The HOPG sample is much weaker than the others, and is not even visible on a linear scale in (a) and is therefore replotted in (b). Because the HOPG signal is relatively weak, a more careful background subtraction was used, with a sloped straight line background used in the signal area, and a linear segment background used in the blocked region. Unlike SWCNTs, HOPG does not have sharp one-dimensional resonances in the density of states and so its REP is relatively featureless, and its intensity variations arise primarily from the instruments spectral characteristics. Between the filter cut offs, the signal is largely flat except for a gradual decrease after 610 nm. Sharp spikes such as that at 612 nm are due to light scattering from particulates on the surface.

The REPs of the chirality sorted samples can be compared to recently published REPs obtained with tunable Raman instruments.^5,23,25^ The (6,5) SWCNT G^+^ band REP is shown in these references, having a principal peak at ~575 nm and a smaller peak or shoulder at the G^+^ phonon energy above the resonance at ~527 nm. References^23,25^ also show the (7,5) SWCNT G^+^ band, with a similar shape, having a principal REP peak at ~651 nm, and a shoulder or peak at the G^+^ phonon resonance above at ~591 nm. References^5,25^ show peak widths of ~0.1 eV FWHM. Reference^23^ has a sharper REP peak of width ~25 meV, possibly narrower because of a more homogenous chemical environment, or more crystalline, or less defective nanotube structure.

The REPs for chirality sorted SWCNTs we see here are broadly consistent. The (6,5) REP peak is at 580 nm or shorter wavelength, cut off by the filter. The (7,5) REP peak appears at ~615 nm but, from the HOPG spectrum it is clear that the sensitivity of the system falls as the wavelength is increased from this point, so this is a lower bound, and it could easily match the 651 nm reported in the other references. Interestingly the (7,5) REP spans the expected range for the two G^+^ REP peaks. We do not observe a distinct high energy peak, but the inflection point near 580 nm may originate from this shoulder. This is consistent with the observation that in these references the low energy REP tends to be well defined and the high energy one much less so. The REPs peaks we observe have the large ~0.1 eV broadening of refs. ^5,25^, not the sharper structure of ref. ^23^. Finally it is interesting that the (6,5) shows a possible small shoulder just before the small wavelength cut-off. Speculatively, this may be the manifestation of the “bundling” REP reported in ref. ^5^. Future experiments with broader scans and/or higher resolution should be able to confirm this.

As we have shown, REMs and REPs can be obtained essentially in real time - orders of magnitude more rapidly than before. (See Supplementary Fig. S6 for examples of usable maps obtained in timescales as short as 5 ms.) The speed of mapping is not just a minor convenience but markedly changes the significance of the technique: it can be used as a routine characterization tool. Furthermore, two independent dimensions of data (vibrational, electronic) are obtained simultaneously, making these maps much more specific fingerprints than conventional one-dimensional RS spectra. They are therefore well suited as inputs into chemometric analysis systems.

Commonly, in RS one has to choose an instrument that operates at an appropriate wavelength. The supercontinuum light with its extreme broadband wavelength range provides great versatility. Moreover, it becomes possible, as demonstrated here at least for some range, to obtain many or even all wavelengths of interest (a “full spectrum”) all at once. Both these aspects help take full advantage of resonance to not only obtain stronger signals, but also obtain expanded spectral fingerprints.

From a photophysical perspective, RRS cross-sections are particularly difficult to evaluate, not only because of the need to characterize instrumental throughput and response, but because, being resonant, they can have sharp, non-linear laser wavelength dependence. The exact resonance wavelength and the breadth of the resonance can be sample environment dependent. In a single fixed wavelength measurement, intensity changes arise from many causes, including this wavelength dependence, and having a continuous REM (in real-time) will help reveal these effects and take them into account.

We believe this method has great potential, and it will benefit from further technical improvements. It is straightforward to extend the bandwidth, or to increase the resolution beyond what we have shown here. We have demonstrated the method on various advanced nanocarbon materials, but it is very general, being relevant to any sample that shows RRS. Given its capabilities, we speculate that full spectrum REM could become a practical tool for chemical analysis and photophysics.

## Supplementary information

Supplementary information.
