# Supplementary material for: Full Spectrum Raman Excitation Mapping Spectroscopy
Source: Sci Rep. 2020 Jun 8;10:9172. doi: 10.1038/s41598-020-65757-9 (PMC7280200; doi:10.1038/s41598-020-65757-9)
Supplement: Supplementary file 1 — Supplementary information. [file 41598_2020_65757_MOESM1_ESM.pdf]

SUPPLEMENTARY INFORMATION for

Full Spectrum Raman Excitation Mapping Spectroscopy

Paul Finnie[1],[2] Jianying Ouyang[1], Jacques Lefebvre[1]

Preparation of Chirality Enriched Nanotubes

Source SWCNTs of the trade name CoMoCAT SG65i were purchased from Sigma-Aldrich (Cat# 773735) and used as the raw source material for (6,5) and (7,5) SWCNTs. The source material for the (9,8) SWCNT was synthesized by chemical vapor deposition (CVD) and generously provided by Sydney University, (Ref. S1). For the polymer wrappings, poly[(9,9-dioctylfluorenyl-2,7-diyl)-alt-co-(6,6'-{2,2'-bipyridine})] (PFO-BPy6,6'), (molecular weight 34kDa, polydispersity 4.3) was purchased from American Dye Source Inc. The poly(9,9-di-n-octylfluorenyl-2,7-diyl) (PFO), molecular weight 54 kDa, polydispersity 2.4) was synthesized in our own laboratories.

To sort, 15.6 mg SWCNT source material was mixed with 15.6 mg of the appropriate polymer in 25 mL of toluene. [Ref. S1, S2]. The polymer was PFO-BPy6,6' for (6,5) SWCNTs and PFO for (7,5) and (9,8) SWCNTs. The mixture was probe-sonicated (Branson Sonifier 250) with a mini-tip of 3/16 inch at an output 30% and a duty cycle of 60% for 30 min, followed by centrifugation at 12500 rpm for 60 min (SS-34 rotor, a relative centrifuge force of 18700 g). This was repeated for multiple cycles to maximize the yield. [Ref. S3] The UV-Vis-NIR absorption of the supernatant was measured by Agilent Cary5000 spectrometer in a quartz cuvette with an optical path of 4 mm to confirm that the samples had the expected absorption spectra for each particular (n,m).

[S1] R. Si, W. Li, H. Wang, D. Su, S. H. Mushrif, Y. Chen, Extraction of (9,8) Single-Walled Carbon Nanotubes by Fluorene-Based Polymers, *Chem. Asian J.* **9**, 868−877. (2014).

[S2] H. Ozawa, N. Ide, T. Fujigaya, Y. Niidome, N. Nakashima, One-pot Separation of Highly Enriched (6,5)-Single-walled Carbon Nanotubes Using a Fluorene-based Copolymer. *Chem. Lett.* **40**, 239−241 (2011).

[S3] J. Ouyang, J. Ding, J. Lefebvre, Z. Li, C. Guo, A. J. Kell, P. R. L. Malenfant, Sorting of Semiconducting Single-Walled Carbon Nanotubes in Polar Solvents with an Amphiphilic Conjugated Polymer Provides General Guidelines for Enrichment. *ACS Nano* **12**, 1910−1919 (2018).

[1] National Research Council Canada, 1200 Montreal Road, Ottawa, Ontario, K1A 0R6
[2] e-mail: Paul.Finnie@nrc-cnrc.gc.ca

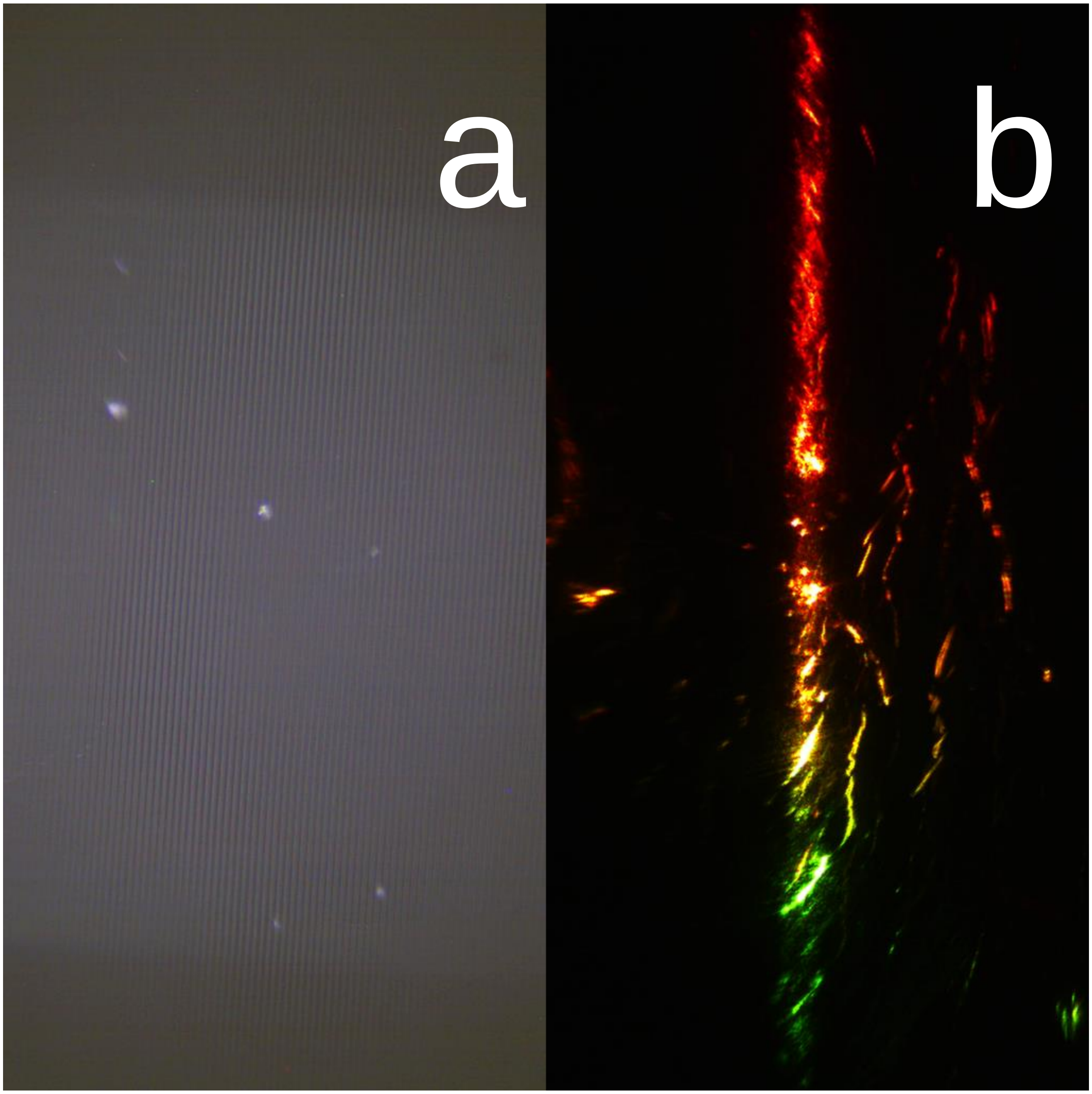

Figure S1. Horizontal Spatial Resolution.

The spatial resolution using the 10× imaging lens and 10× focusing lens as determined by a color webcam. (a) A “Ronchi ruling” (Edmund Optics), which is a periodic array of lines on glass, here at 100 lines/mm pitched, imaged in white light illumination (b) The “rainbow” line illumination imaged on a graphite (HOPG) sample. The width of the rainbow line in the red region is ≈43 μm. Light from scattering particles and other features on this surface is also visible.

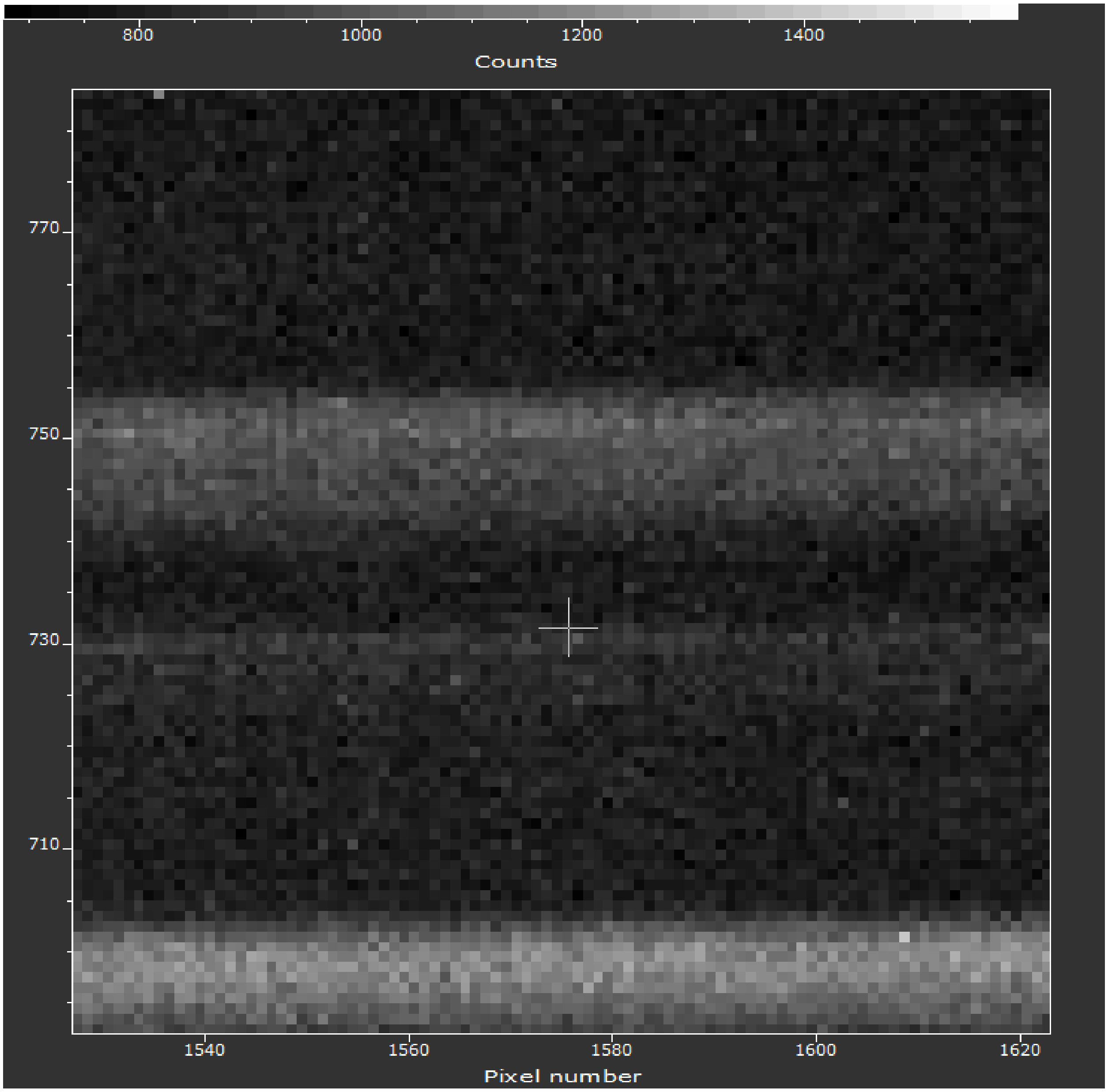

Figure S2. Vertical Spatial Resolution

The vertical spatial resolution is shown to be limited by the pixel size on the camera. This is a raw screen capture of a contaminated area of a graphene surface. The cross-hair shows a feature which is a single pixel high. It appears as a horizontal line because it is spectrally dispersed.

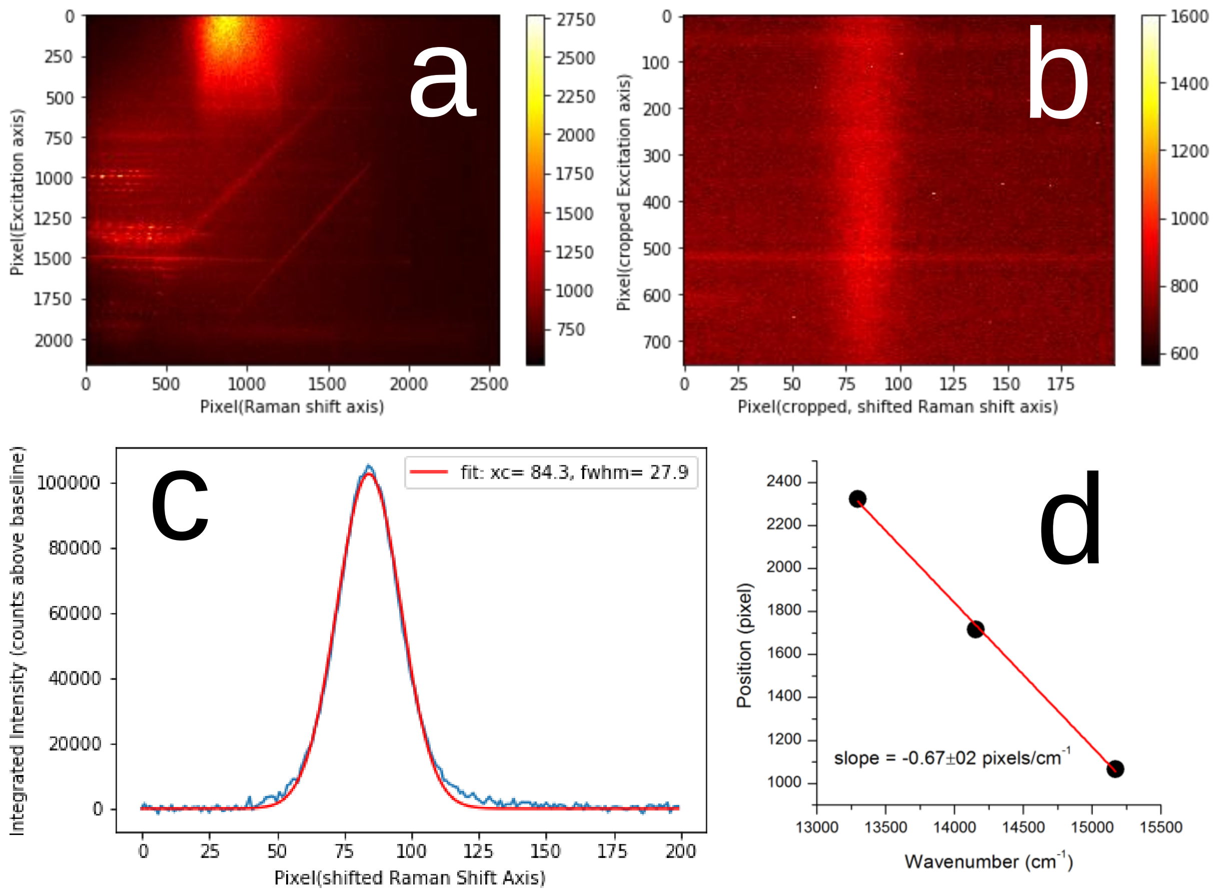

Figure S3. Spectral Resolution. (a) An one minute acquistion from a highly oriented pyrolytic graphite (HOPG) sample. The G band is the narrow diagonal line in the middle of the plot. HOPG is known to have a fairly sharp (~10 - 15 cm^{-1} wide) G band near 1580 cm^{-1}.[S5] In (b) each vertical slice has been translated by a linearly increasing offset, so that the G band line is vertical. The image is cropped to show only the G band. In (c) the vertical scans are summed to a single curve covering the entire range. This is fitted with Python (numpy and scipy.optimize) to a single Gaussian peak (fit) having full width half maximum (fwhm) of ~28 pixels. The dispersion of the imaging grating on the camera (horizontally here) is ~ 0.67 pixels/cm^{-1}, as determined by the bandpass filters (d), so the fwhm corresponds to ~41 cm^{-1}, which is larger than the real linewidth of HOPG.

Pixels on the camera are 6.5 μm wide, so 28 pixels corresponds to ~180 μm on the camera. The imaging was with a 10× objective. Since a 75 mm tube lens was used, rather than a 200 mm tube lens, the magnification is smaller by the ratio of these lengths, namely, so it is effectively ~3.8×. So on the camera this represents ~47 μm on the sample. This matches the spatial width of the illumination line on the sample. Therefore, thus indicates that spectral resolution of the Raman shift is limited here by the spatial width of the illuminating strip.

[S5] A. Jorio, M. Dresselhaus, R. Saito, G. F. Dresselhaus, *Raman Spectroscopy in Graphene Related Systems*, Wiley-VCH, Weinheim 328 (2011).

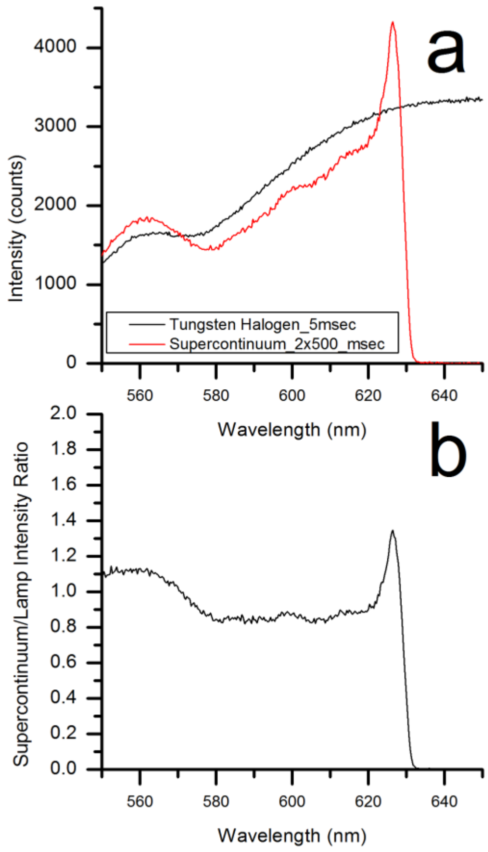

Figure S4. The wavelength dependence of the illumination. The microscope objective focused, grating dispersed supercontinuum beam was scattered off a glass diffuser, collected by a collimating lens and delivered to an optical spectrometer (Ocean Optics USB2000) by optical fiber (Ocean Optics P200-2-VIS-NIR). (a) The spectrum of a long life tungsten halogen bulb (Ocean Optics LS-1-LL), following the same optical path, is shown in black. The product documentation indicates the bulb should approximate blackbody (estimated at temperature ~2800K). The spectrum of scattered light from the supercontinuum light source is shown in red, after all excitation optics. (b) The ratio of the supercontinuum illumination to lamp illumination (normalized)

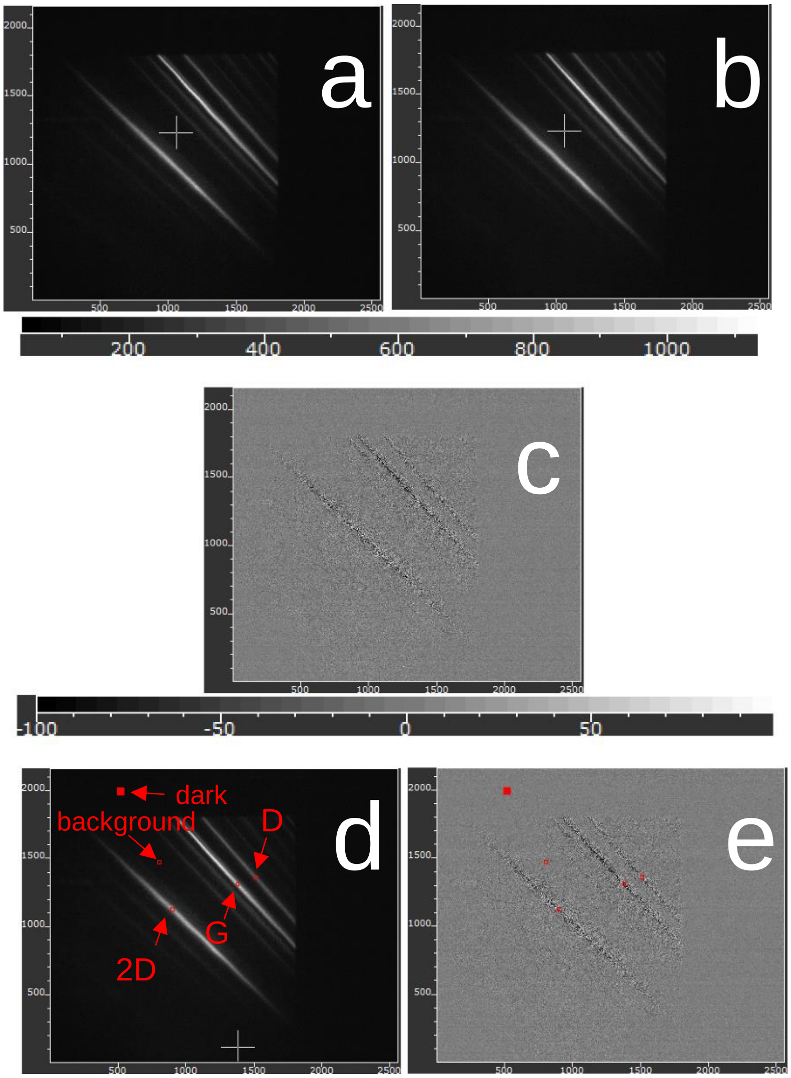

Figure S5. Signal-to-Noise

This figure graphically demonstrates a quantitative estimate of the signal-to-noise. This is an adaptation of the procedure outlined in Ref. [S4] used to estimate signal-to-noise from conventional Raman spectra.

Figures (a) and (b) show the raw REM data for two otherwise identical 500 ms acquisitions, taken minutes apart, for the (7,5) SWCNT sample. Here, the incident power was ~10 mW over the band ~540 nm to ~630 nm. The *x*-axis is in pixels, corresponding to emission wavelength and the *y*-axis is also in pixels, corresponding to excitation wavelength, the grayscale below is in counts (counts in analog to digital units [ADU]). Figure (c) shows the difference in the two images, with the corresponding scale bar shown beneath, calculated by the Andor Solis software. A 25×25 pixel area was selected to be wide enough (in *x*) to include the peak of the Raman band, but small enough (in *y*) to simulate "monochromatic" excitation. Panel (d) is the same as panel

(a) but with the 25×25 pixel regions of interest identified by boxes, labelled by the particular Raman band (D, G, 2D) or physical origin (dark = not illuminated, background = illuminated, but no defined Raman band.) (e) shows the same regions of interest on the difference spectrum. The average intensity in the ROI for one raw image was calculated by the Andor software. The standard deviation of the difference image was calculated from for the same ROI. The standard deviation of the peak height is the standard deviation of the difference divided by $\sqrt{2}$.

[S4] McCreery, R. L. Raman spectroscopy for chemical analysis, *Chemical Analysis: A Series of Monographs of Analytical Chemistry and Its Applications*, J. D. Winefordner, Ed., Wiley-Interscience, New York **157,** 49-71 (2000).

Band	Average Intensity (counts)	Average Intensity above fixed Background (counts)	Standard Deviation Of Difference (counts)	Standard Deviation Of Peak Height (counts)	Signal-to-Noise Ratio
**G**	754	630	49	35	18
**D**	365	241	33	24	10
**2D**	655	531	49	35	15
**background**	124	-	12	8	-
**dark**	100	-24	6	4	-

Table S1. Calculated Signal-to-Noise

Following Ref. [S4], we calculate the Signal-to-Noise Ratio (SNR) defined as the inverse of the relative standard deviation of the peak height. To be more specific, we take average peak intensity in counts (i.e. first column of numbers in Table S1) as the effective peak height $\langle P \rangle$. This actually underestimates the true peak height since the illumination linewidth in pixels is comparable to the Gaussian FWHM in pixels. That is, the true Gaussian peak height is somewhat higher than this average quantity. Using a constant fixed background B, the background corrected peak intensity above background is $\langle S \rangle = \langle P \rangle - B$ (i.e. second column of numbers in Table S1). By taking two otherwise identical spectra and subtracting them, we get a spectrum of the noise alone. We define the standard deviation of the peak height (σ) from this noise spectrum (second last column of table S1). This is obtained from the same pixels used to determine the peak intensities. Then following Ref. [S4], the signal-to-noise ratio (SNR) is given by $SNR = \langle S \rangle / (\sigma/\sqrt{2})$. (This calculated value is shown in the last column of Table S1). The resulting SNR is ≈18 for the G^+ band of the (7,5) sample.

The SNR can be increased by using more excitation wavelengths (more of the *y*-axis) for the band in same aquisition, or by increasing the acquisition time.

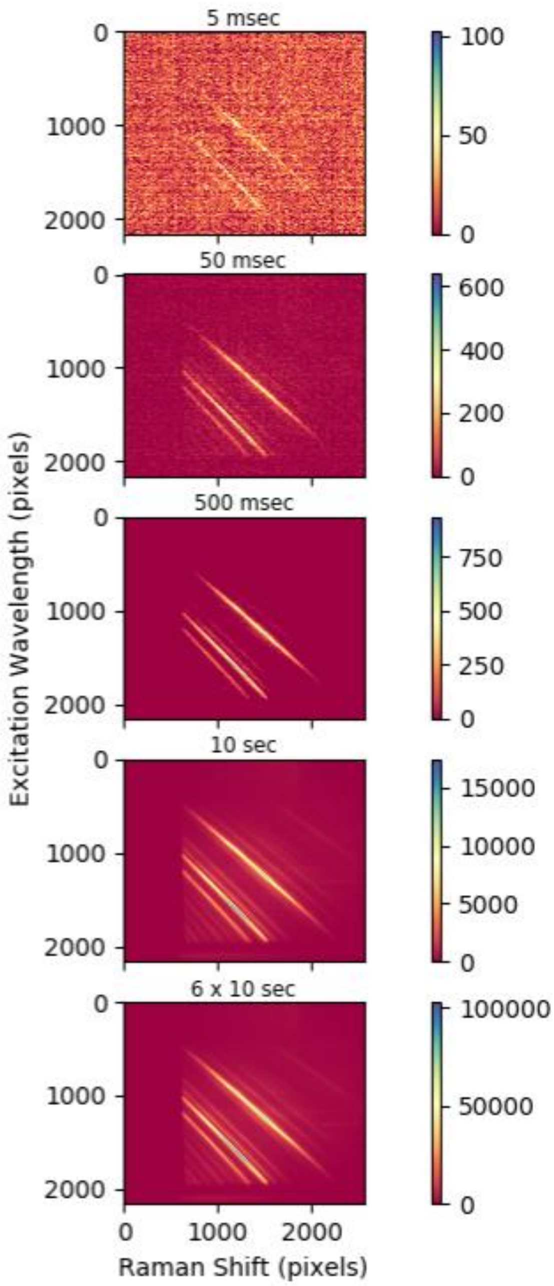

Figure S6. Qualitative Signal-to-Noise: Different Integration Times

The (7,5) sorted SWCNT sample is shown for 5 different exposure times, and so improving signal-to-noise, from top to bottom: 5 ms, 50 ms, 500 ms, 10 s, six integrations of 10 s summed. A reasonably strong signal is obtained in just 50 ms, and even at 5 msec the stronger bands are still above the noise level.

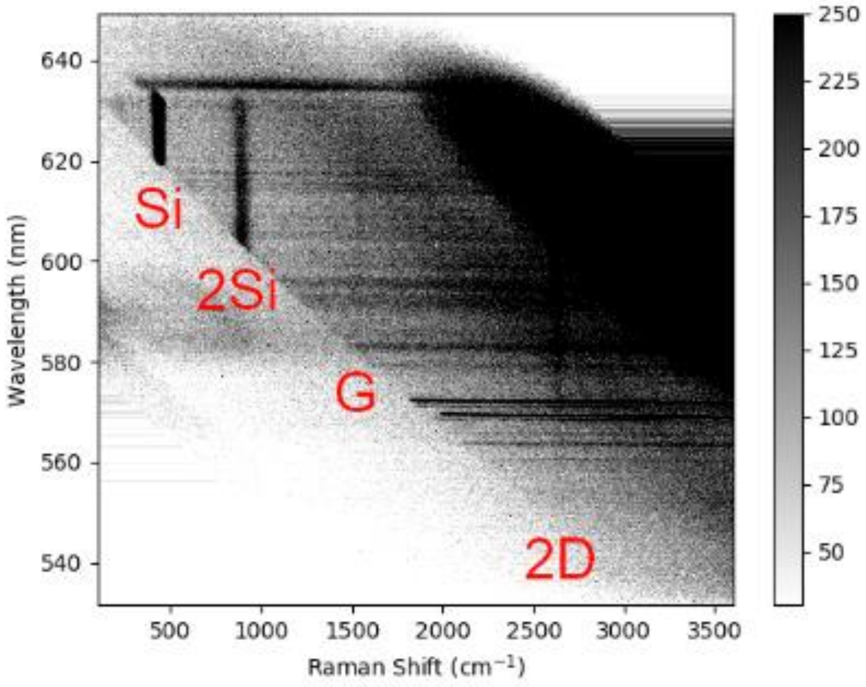

Figure S7. The G band of Graphene

This map is plotted on an inverted gray scale (black is most intense) to show that the G band is visible for the graphene sample. The bands shown are from the silicon substrate (Si), the second order of the silicon substrate (2Si) the 2D band and the faint, but visible G band. This was a one minute acquisition made by accumulating six 10s exposures.
